# Neuronal mechanisms of motor learning and motor memory consolidation in healthy old adults

**DOI:** 10.1007/s11357-015-9779-8

**Published:** 2015-05-09

**Authors:** K. M. M. Berghuis, M. P. Veldman, S. Solnik, G. Koch, I. Zijdewind, T. Hortobágyi

**Affiliations:** Center for Human Movement Sciences, University Medical Center Groningen, University of Groningen, A. Deusinglaan 1, Groningen, 9700 AD The Netherlands; Motor Control Laboratory, Department of Kinesiology, Pennsylvania State University, State College, PA USA; University School of Physical Education, Wroclaw, Poland; Laboratorio di Neurologia Clinica e Comportamentale, Fondazione Santa Lucia IRCCS, Rome, Italy; Department of Neuroscience, University Medical Center Groningen, University of Groningen, Groningen, The Netherlands; Faculty of Health and Life Sciences, Northumbria University, Newcastle-upon-Tyne, UK

**Keywords:** Motor practice, Attentional control, Transcranial magnetic stimulation, Corticospinal excitability, Short-interval intracortical inhibition, Elderly

## Abstract

It is controversial whether or not old adults are capable of learning new motor skills and consolidate the performance gains into motor memory in the offline period. The underlying neuronal mechanisms are equally unclear. We determined the magnitude of motor learning and motor memory consolidation in healthy old adults and examined if specific metrics of neuronal excitability measured by magnetic brain stimulation mediate the practice and retention effects. Eleven healthy old adults practiced a wrist extension-flexion visuomotor skill for 20 min (MP, 71.3 years), while a second group only watched the templates without movements (attentional control, AC, *n* = 11, 70.5 years). There was 40 % motor learning in MP but none in AC (interaction, *p* < 0.001) with the skill retained 24 h later in MP and a 16 % improvement in AC. Corticospinal excitability at rest and during task did not change, but when measured during contraction at 20 % of maximal force, it strongly increased in MP and decreased in AC (interaction, *p* = 0.002). Intracortical inhibition at rest and during the task decreased and facilitation at rest increased in MP, but these metrics changed in the opposite direction in AC. These neuronal changes were especially profound at retention. Healthy old adults can learn a new motor skill and consolidate the learned skill into motor memory, processes that are most likely mediated by disinhibitory mechanisms. These results are relevant for the increasing number of old adults who need to learn and relearn movements during motor rehabilitation.

## Introduction

Even healthy aging is associated with an up to 50 % reduction in the number and diameter of motoneuron axons, a decrease in number of large-diameter axons, slowing of peripheral nerve conduction, impaired sensory fiber function, prolongation of reflex latencies, and a loss and subsequent remodeling of motor units (Aagaard et al. 2010). Modifications in the peripheral nervous system are accompanied by substantial and functionally relevant reductions in gray matter volume in the primary motor, somatosensory cortices, and the cerebellum (Goble et al. 2009; Good et al. 2001; Salat et al. 2004; Ward and Frackowiak 2003). In addition to cortical atrophy, there are quantitative and qualitative changes in white matter structure and integrity (reviewed in Seidler 2010; Seidler et al. 2010). Such and other age-related changes in the neuromuscular system and a general reduction in motor activity make voluntary movements weak, slow, unsteady, and inaccurate (Aagaard et al. 2010; Clark and Fielding 2012; Spirduso 2010). With regard to the relatively well-characterized age-related changes in neuromuscular properties, a more contentious issue is whether or not healthy old adults can learn and retain new motor skills. Understanding the mechanisms of how and if age affects the ability to learn and relearn motor skills is especially relevant because, with increasing age, more and more old adults receive movement rehabilitation that includes the learning and relearning of movements impaired by specific comorbidities (Krakauer 2006), as, for example, is the case after a stroke (Hummel et al. 2009). In addition, a better understanding of how healthy old adults learn and relearn a novel motor skill is important because many old adults must operate and manipulate new electronic devices and need to acquire motor skills in new jobs (Czaja and Sharit 2009; Zimerman et al. 2013).

Despite the many unfavorable age-related changes in neuromuscular function and brain structures involved in motor learning, results from a group of studies provide evidence that age may not necessarily impair the ability to acquire novel motor skills (Brown et al. 2009; Coats et al. 2014; Roig et al. 2014; Swinnen 1998; Zimerman et al. 2013). For example, old and young adults, practicing a visuomotor tracking task for 18 min, showed similar, about 23 %, performance gains (Cirillo et al. 2011). However, another group of studies reported that the ability to learn new motor skills in a single training session decreases with age (Coats et al. 2014; Swinnen 1998; Zimerman et al. 2013). To illustrate, the learning rate of a bimanual coordination pattern with 90° phase offset between the limbs is smaller in seniors compared with adolescents (Swinnen 1998). Finally, there is some evidence suggesting that performance gains in reaction time are actually superior in old compared with young adults (Brown et al. 2009).

In addition to the immediate performance gains, another important element of motor learning is the ability to retain and recall the previously acquired motor skills. Motor memory consolidation is the stabilization of memory traces following the initial online motor learning or acquisition period and can result in increased resistance to interference or even an improvement in performance after an offline period (Janacsek and Nemeth 2012). There is some evidence for an age-related decline in motor memory consolidation because old adults were able to stabilize the learned reaction time skills at the retention test 24 h after the first training session (retention gain = −4.5 ms, *p* > 0.05), whereas young subjects showed not only stabilization but further improvements in the retained skills in the offline period (retention gain = 36.8 ms, *p* < 0.01) (Brown et al. 2009). In other studies, reaction time improved after motor practice during the 12-h offline period with greater gains in young compared with old adults (Nemeth et al. 2010; Nemeth and Janacsek 2011). Young adults also showed improvements at 24-h and 1-week retention test, whereas old adults did not (Nemeth et al. 2010; Nemeth and Janacsek 2011). Furthermore, a recent study showed that memory consolidation of a ballistic wrist flexion skill is impaired with aging (Roig et al. 2014), and finally, sequence-specific knowledge decreased between sessions in old, but it stayed stable in young adults, suggesting weaker consolidation of sequence-specific knowledge in the elderly (Nemeth and Janacsek 2011). However, we must note the wide variation in methods that these studies used to examine motor learning and motor memory consolidation in aging.

There is a paucity of data concerning the underlying neuronal mechanisms involved in motor learning and motor memory consolidation in old adults. A transcranial magnetic stimulation (TMS) study compared corticomotor excitability and short-interval intracortical inhibition (SICI) between young and old adults after 300 rapid thumb abduction movements (Rogasch et al. 2009). Old (124 %) compared with young (177 %) adults achieved lower gains in motor performance. Corticomotor excitability increased after motor practice in young but not in old subjects, and motor practice did not modify SICI in either age group. Practice of a complex visuomotor task in the form of index finger ab- and adduction improved task accuracy similarly in both age groups (7–24 % range) with an increase in corticospinal excitability and reduction in SICI independent of age (Cirillo et al. 2011). None of these studies examined motor learning, motor memory consolidation, as well as indices of neuronal mechanisms in combination in healthy older adults.

Changes in corticospinal excitability (CSE) measured at rest presumably reflect changes in long-term potentiation-like mechanisms involved in motor learning (Butefisch et al. 2000; Muellbacher et al. 2002; Sawaki et al. 2002). However, no studies have examined if changes in CSE after motor learning would also occur during task performance in old adults. Measurements at rest and during task performance seem intuitively and mechanistically warranted because these could reflect the activation of different portions of the motoneuron pool and also changes in the input–output gain of individual motoneurons or at the level of the motoneuron pool (Di Lazzaro et al. 1998a; Smith et al. 2011). In addition, SICI is a GABA-A-mediated inhibition that occurs in primary motor cortex (M1) circuits (Di Lazzaro et al. 1998b; Kujirai et al. 1993), and its reduction is associated with the induction of long-term potentiation (Floyer-Lea et al. 2006). Measurement of SICI not only at rest, as it has been done in all previous motor learning studies using TMS, but also during the task itself would add to the mechanistic understanding of motor learning by increasing the specificity of measurements. Based on the mixed results reported previously concerning the changes in CSE and SICI at rest in young and old adults after motor learning (Cirillo et al. 2010, 2011; Rogasch et al. 2009), we favor the hypothesis that measurements of neuronal excitability when the muscle is active (i.e., during the task or a muscle contraction) are more sensitive and specific to motor learning than the same tests performed at rest after motor practice. This is because, after motor skill learning, there is an increase in brain activation in secondary motor areas, for example, premotor and supplementary motor areas (for a review, see Dayan and Cohen 2011), making it likely that neuronal excitability measurements during contraction but not at rest would represent activity of secondary motor areas upstream M1.

The aim of this study was to determine the magnitude of motor learning and motor memory consolidation in healthy old adults and examine, for the first time, if specific metrics of motor cortical and corticospinal function measured by TMS mediate the practice and retention effects. Because motor learning is known to rely on attentional resources (Dayan and Cohen 2011; McNevin et al. 2000; Saucedo Marquez et al. 2011), our experimental approach controlled for the attentional load associated with motor practice, an element absent in previous studies.

## Methods

### Subjects

Twenty-two healthy older adults volunteered to participate in this study (14 men and 8 women; age, 70.9 ± 2.9 years; height, 1.74 ± 0.09 m; weight, 78.9 ± 15.3 kg; body mass index, 26.1 ± 5.3 kg/m^2^). We evaluated subjects’ health status using the Groningen Activity Restriction Scale (GARS), a reliable and valid test of disability in Activities of Daily Living (ADL) or Instrumental ADL (IADL) (Kempen et al. 1996). We assessed subjects’ cognitive health with the Mini-Mental State Examination (MMSE; Folstein et al. 1975). Handedness was evaluated with the Edinburgh handedness inventory (Oldfield 1971). Subjects were excluded from the study if they suffered from neurological conditions, took medications influencing nerve conduction velocity, and had contraindications for the use of TMS, a pacemaker, metal in the brain or skull, and had uncorrected vision (Rossi et al. 2009). Subjects were also excluded if they had pain or movement constrictions in their right arm or hand. Subjects were asked not to consume coffee or tea an hour before the start of the experiment on each of the two testing days. Subjects signed an informed consent document, approved by the Medical Ethical Committee of the University Medical Center Groningen.

### Procedure

**Fig. 1:**
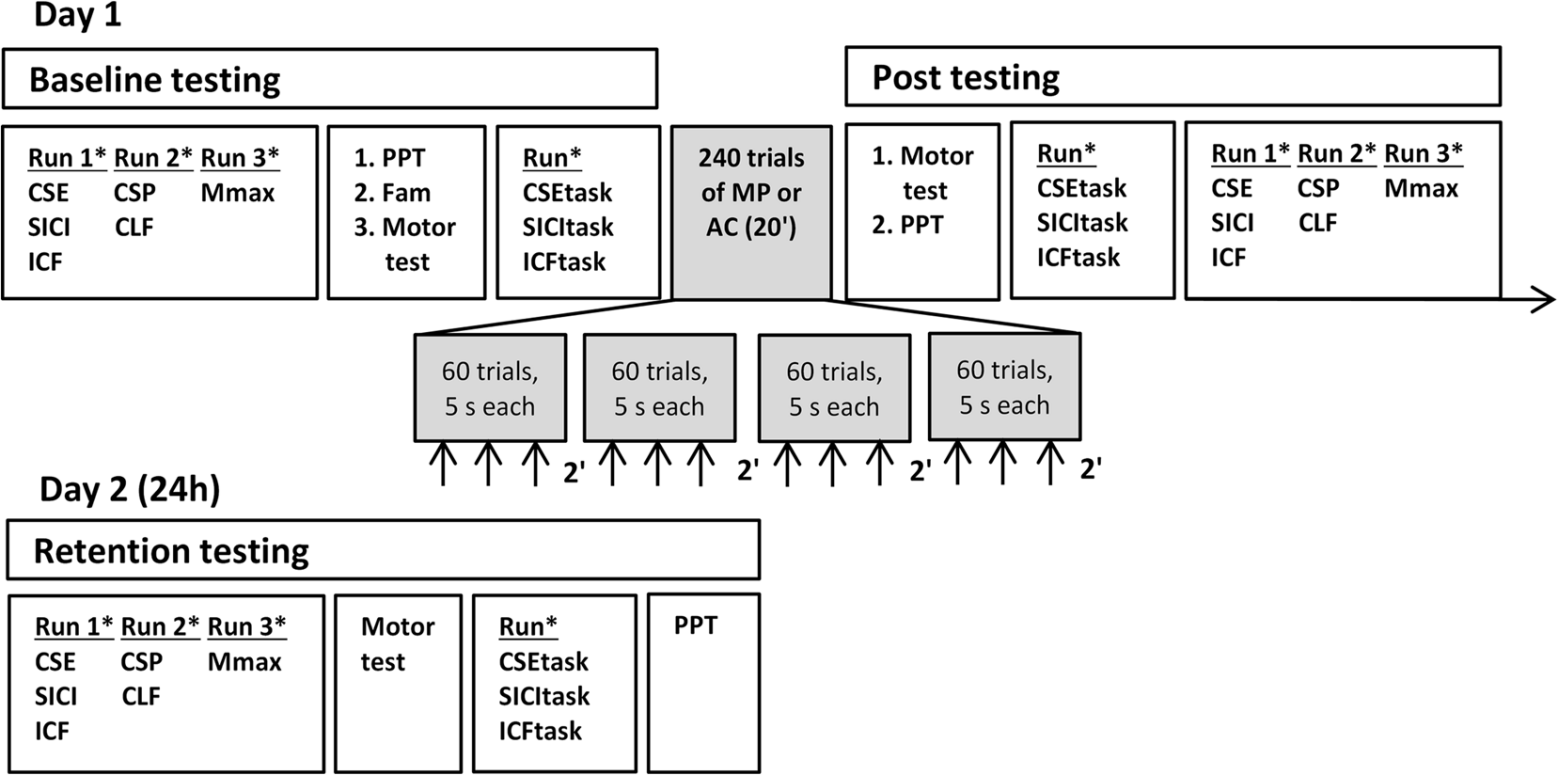
The experimental design consisted of the pre- and posttests on day 1 and a retention test on day 2. *Upward directed arrows* indicate the time when subjects performed a counting task to control for attentional drift. The order of the runs within a block and the order of the pulses within a block were randomized (*asterisk*). *CSE* corticospinal excitability, *SICI* short-interval intracortical inhibition, *ICF* intracortical facilitation, *CSP* cortical silent period, *CLF* contralateral facilitation, *Mmax* maximal compound action potential, *PPT* Purdue pegboard test, *Fam* familiarization, *CSEtask* corticospinal excitability during task, *SICItask* short-interval intracortical inhibition during task, *ICFtask* intracortical facilitation during task, *MP* motor practice, *AC* attentional control

Subjects were randomly assigned to one of two groups: motor practice group (MP) or attentional control group (AC). Testing procedure consisted of a pre-, post-, and retention test (Fig. 1). Pre- and posttests were performed on day 1, and the retention test was performed 24 h later on day 2. To control for variation in responses to TMS due to a diurnal effect, the retention tests were administered within ±30 min of the time when the pretest was administered 24 h earlier, during the day between 9 am and 3 pm. The design included a 24-h retention interval, categorized normally as a delayed test (Kantak and Winstein 2012). The pretest consisted of TMS measurements at rest and during the motor task, peripheral nerve stimulation that determined the maximal compound action potential (Mmax), hand function test, and the baseline assessment of visuomotor skill. TMS parameters included corticospinal excitability at rest (CSE) and during the visuomotor task (CSEtask), short-interval intracortical inhibition at rest (SICI) and during the visuomotor task (SICItask), intracortical facilitation at rest (ICF) and during the task (ICFtask), cortical silent period (CSP), and contralateral facilitation (CLF) at 20 % of maximal voluntary contraction (MVC). After the pretest, one of the two interventions was performed for a period of 20 min: Subjects either performed MP or AC. Subjects in MP performed the visuomotor task during the intervention period. The duration of the intervention was based on previous data suggesting that such a practice period is sufficient to reliably produce fast motor learning (Cirillo et al. 2011; Rogasch et al. 2009). Because motor learning is known to involve strong attentional elements (Dayan and Cohen 2011; McNevin et al. 2000; Saucedo Marquez et al. 2011), our design also included a group in which we assessed the magnitude of learning produced by attention to the task. Subjects in AC focused, during the intervention period, their attention on the visuomotor templates that appeared on the monitor but did not perform any movements. Instructions were as follows: “Follow the template only with your eyes but not with your hand.” The posttest was a repeat of the pretest in both groups. On day 2, sleep quality and quantity of the last month and last night were determined using the Pittsburgh Sleep Quality Index (Buysse et al. 1989). In addition, we repeated the pretest measurements to quantify the retention of motor memory traces and to determine the long-lasting changes in measures of neuronal excitability.

In a control experiment conducted in additional five healthy, right-handed old adults (age, 69.8 ± 3.83 years), we examined the possibility that only familiarization of subjects with the motor task could produce learning and affects also retention. We also wished to quantify the variability in the TMS data by repeating these measurements three times. These subjects performed the same protocol as did the subjects in the main experiment, but instead of motor practice and attentional control, they sat for 20 min and read newspapers, using their left hand to turn pages.

### Behavioral testing and motor practice

Subjects sat comfortably in a chair without armrests approximately 90 cm in front of a laptop computer’s monitor (diagonal distance, 39.6 cm). Their right forearm was fixed in a padded manipulandum in a neutral wrist position, the thumb pointing upwards. The center of the wrist joint was aligned with the axis of the manipulandum that confined wrist motion to flexion and extension. The left arm was resting on a table covered with soft material in a pronated position. The knees were flexed 90°, and the feet were flat on the floor.

As reported previously, we used a visuomotor task for behavioral testing and also for the motor practice intervention, consisting of template tracking (Cirillo et al. 2011; Jensen et al. 2005; Perez et al. 2004). Subjects were asked to match the template as accurately as possible by flexing and extending the right wrist. The template appeared on the monitor, proceeded from left to right, and changed direction that prompted wrist extension (template up) and flexion (template down). The background on the monitor was dark blue and contained a hairline-thick light blue-colored grid. The template appeared in white, and the subject’s performance line appeared in green color in high resolution.

Trials used for testing subjects’ visuomotor skill consisted of six templates of different patterns. Templates were scaled to each subject’s wrist range of motion. Trials used for the interventions also consisted of six different template patterns. Templates used for the interventions and the templates used to assess learning were different but were of similar difficulty as quantified by the number of turns. There were one or two turns within each template, i.e., changes in direction (mean, 1.33 ± 0.49). The order and duration of the templates were randomized but was the same for each subject at the three tests. The duration of the templates varied between 4, 5, or 6 s (mean, 4.99 ± 0.82 s).

Prior to testing, subjects performed three familiarization trials. Next, they completed 12 pretest trials to establish baseline. After this pretesting, MP completed 4 blocks of 60, a total of 240 trials. After every 15 trials, subjects in both groups were asked to count backwards by seven to minimize attentional drift. Between training blocks, subjects in both groups rested for 2 min. After the interventions, subjects repeated the same 12 trials used in the pretest to assess the magnitude of motor learning. On day 2, a retention test containing 12 trials was administered.

### Hand function

In order to determine if the acquisition and/or motor memory consolidation of the visuomotor skill transferred to a nonpracticed motor task, i.e., a task variant, the Purdue pegboard test was administered at baseline and after motor practice and attentional control on day 1 and also on day 2 during the retention test (Tiffin and Asher 1948). The Purdue pegboard test reliably measures gross motor movements of the arms, hand, and fingers, and fine motor dexterity (Desrosiers et al. 1995; Thomas 2014).

### EMG recording

Subject’s skin was prepared for electromyography (EMG) by shaving, scrubbing with fine sandpaper, and cleaning the skin with alcohol to minimize noise in the EMG signal. EMG was recorded in the left and right flexor carpi radialis (FCR) and left and right extensor carpi radialis (ECR) and using 37 × 27 × 15mm, <15 g, wireless, preamplified (909×) parallel-bar sensors, affixed to the skin with a four-slot adhesive skin interface (Trigno, Delsys Inc., Natick, MA, USA). The electrodes recorded with a bandwidth of 20–450 Hz, channel noise <0.75 μV, and common mode rejection ratio >80 dB. EMG activity was sampled at 4 kHz. Signals were acquired online and stored by software installed on a personal computer for offline analysis (Power 1401 and Signal, Cambridge Electronics Design, Cambridge, UK).

### Transcranial magnetic stimulation

Single- and paired-pulse TMS measurements were performed with two Magstim 200 magnetic stimulators (Magstim Company Ltd, Dyfed, UK). A figure of eight coil (loop diameter, 90 mm) was connected to BiStim^2^ stimulators and held over the optimal stimulation spot of the left motor cortex to elicit motor-evoked potentials (MEPs) in the right ECR with the handle pointing backwards at ∼45° away from the sagittal plane. To ensure consistent coil position during the experiments, the optimal point, the hot spot, for stimulating the right ECR, was marked on a cloth cap that the subjects wore. Resting motor threshold (RMT) was defined as the minimum intensity (percent stimulator output) where five out of the 10 trials evoked an MEP in the right ECR with amplitude ≥50 μV (Kujirai et al. 2006; Rossini et al. 1994). Additionally to RMT, in nine subjects, active motor threshold (AMT) was measured, defined as the minimum intensity (percent stimulator output) where five out of the 10 trials evoked a MEP in the right ECR with amplitude ≥200 μV and above-background EMG signal during isometric contraction of the right ECR at 10 % MVC (Rothwell et al. 1999).

CSE, SICI, and ICF were determined at rest. Test pulse was set at 120 % RMT, and conditioning pulse was set at 80 % RMT (Kujirai et al. 1993). The interval between the paired pulses for determining SICI and ICF were, respectively, 2 and 10 ms (Kujirai et al. 1993). Subjects received a total of 30 pulses, randomized 10 single pulses, 10 paired pulses with 2-ms interval, and 10 paired pulses with 10 ms interval.

CSE (Barthelemy et al. 2012; Forman et al. 2014; Petersen et al. 1998a; Sidhu et al. 2012), SICI, and ICF were also measured during the visuomotor task (CSEtask, SICItask, and ICFtask) in nine subjects. Subjects completed 30 trials of the visuomotor task. These trials started with a flexion followed by an extension movement but still had an element of difficulty because there were five different templates appearing in a random order. During the extension phase of the trial as the wrist passed at 8° extension, subjects received randomized 10 single pulses, 10 paired pulses with 2-ms interval, and 10 paired pulses with 10-ms interval. Conditioning pulse was set at 70 % AMT and test pulse at 120 % AMT (Ortu et al. 2008).

CSP and CLF were measured to determine motor cortical inhibition and facilitation during weak muscle contraction specific to the task. Subjects received 15 TMS pulses at 120 % RMT. The first five pulses subjects had both arms in rest, but during the next 10 pulses, subjects performed an isometric contraction at ±8° into wrist extension at 20 % MVC. CSP is the interruption of ongoing EMG activity after a TMS pulse is given Kojima et al. (2013).

### Peripheral nerve stimulation

Mmax was defined as the maximal peak-to-peak amplitude of the M-wave as a response to electrical stimulation of the right radial nerve above the elbow. An electrical stimulator delivered the 0.5-ms-long square-wave stimulus (DS7A, Digitimer Ltd, Welwyn Garden City, UK). The stimulation intensity was increased until the peak-to-peak amplitude of the M-wave did not increase any further and then stimulation intensity was raised by 20 % to ascertain Mmax.

### Data analysis

Matlab R2011a was used to analyze the behavioral data, i.e., the performance on the visuomotor task, and the CSP data (The Mathworks Inc., Natick, MA, USA). Visuomotor skill was determined by calculating the mean error of the subject’s wrist joint position from the white preprogrammed template. The first second of the behavioral data was discarded because it contained errors associated with reacting to the appearance of the template. CSP onset, offset, and duration were determined using an adjusted version of the Teager Kaiser energy operator (TKEO), a highly effective method used to determine the boundaries of an EMG burst (Solnik et al. 2008). Signal 5.04 was used to analyze the remaining TMS parameters. Peak-to-peak amplitudes of MEPs were calculated in order to determine CSE, CSEtask, SICI, SICItask, ICF, ICFtask, and CLF. CSE and CSEtask were expressed by the MEP amplitude as a percentage of Mmax. SICI and ICF at rest and during the task were expressed by the conditioned MEP as a percentage of the test MEP. CLF was defined as the mean peak-to-peak MEP amplitude of the trials with 20 % MVC expressed as a percentage of the mean peak-to-peak MEP amplitude of the trials in rest. The background EMG activity was calculated as the mean rectified EMG activity in the period 70 ms before the TMS test pulse.

### Statistical analyses

Data are reported as mean ± SD. Two-way repeated measures analysis of variances (ANOVA) was performed to determine the effects of intervention (MP, AC; between-subjects factor), time (baseline, posttest, retention at 24 h; within-subjects factor), and interactions of intervention and time on visuomotor skill, Purdue Pegboard performance, Mmax, RMT, AMT, CSE, CSEtask, SICI, SICItask, ICF, ICFtask, CLF, and CSP. When there was a between-group difference at baseline, an analysis of covariance (ANCOVA) was performed, using baseline values as a covariate. Tukey’s post hoc analysis was performed to determine the means that were different from one another. In the control experiment, we performed one-way repeated measures ANOVAs to determine if there was a main effect of time in each dependent variable.

In order to determine if baseline values and changes in visuomotor skill were associated with Purdue Pegboard performance and TMS variables (CSE, CSEtask, SICI, SICItask, ICF, ICFtask, CLF, and CSP), Pearson’s correlations were computed. For all analyses, we set the level of significance at *p* < 0.05.

## Results

Table 1 shows that the 11 subjects (7 M and 4 F) in MP and AC were similar in age, MMSE, laterality score, GARS, PSQI, and the quantity and quality of sleep the night before testing. The 11 subjects (7 M and 4 F) in AC vs. MP were somewhat heavier and taller.

**Table 1:** Characteristics of subjects in the motor practice group (MP, *n* = 11) and attentional control group (AC, *n* = 11)

Variable	MP mean (±SD)	AC mean (±SD)
Age (years)	71.3 (3.35)	70.5 (2.50)
Mass (kg)	73.3 (9.34)	84.5 (18.32)
Height (m)	1.71 (0.10)	1.77 (0.07)
BMI (kg/m^2^)	24.9 (1.92)	27.4 (7.20)
MMSE	28.7 (1.74)	29.4 (1.00)
GARS	18.4 (1.21)	18.1 (0.3)
Laterality quotient	0.91 (0.09)	0.96 (0.08)
PSQI	5.2 (4.29)	5.0 (3.97)
Quantity of sleep (h)	6.7 (1.69)	7.2 (0.94)
Quality of sleep^a^	1	1

*BMI* body mass index, *MMSE* Mini Mental State Examination (>27 cognitively healthy), *GARS* Groningen Activity Restriction Scale (18–72, the higher the score, the higher the activity restriction), *PSQI* Pittsburgh Sleep Quality Index (lower score is higher quality of sleep in last month), quantity of sleep in hours the night before retention testing, quality of sleep on a scale from 0 (best) to 3 (worst) in the night before retention testing

^a^Instead of mean (±SD), the modus is shown for the results of this 4-point Likert scale

### Behavioral data

**Fig. 2:**
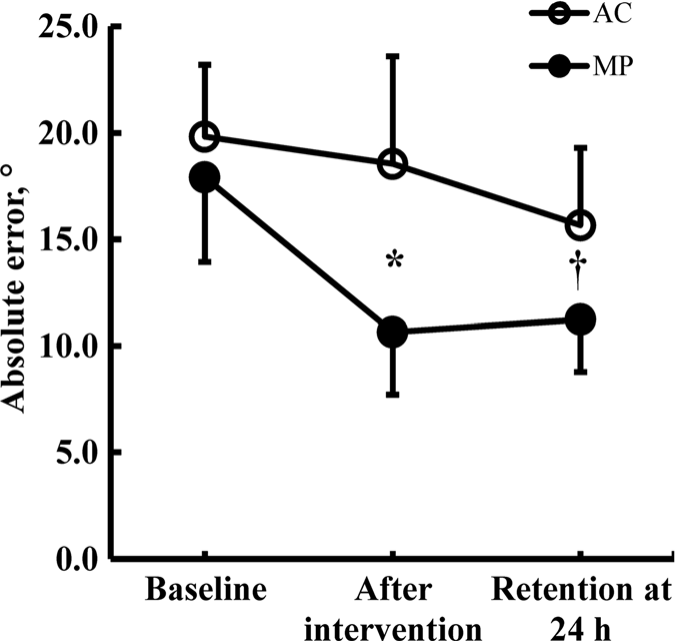
Motor learning data. The magnitude of error in the two groups was similar at baseline. After active motor practice (*filled symbols*), the magnitude of error was significantly lower compared with baseline and compared with attentional control (*open symbols*, *asterisk*). After 24 h, the magnitude of error after attentional control was lower compared with baseline but greater than after motor practice (*dagger*). *Vertical bars* denote ±1 standard deviation

Figure 2 shows the group × time interaction in the amount of error [*F* (2, 40) = 12.3, *p* = 0.000]. With the two groups producing similar amount of error at baseline (difference, 1.9°, n.s.), after intervention, the reduction in error from baseline to posttest was 40 % or 7.3° in MP (*p* < 0.05) and 6 % or 1.3° in AC. At retention, MP maintained the posttest error level (0.6° more error, n.s.) while, relative to baseline, the error in AC decreased by 16 % or 2.9° (*p* < 0.05, relative to baseline). From baseline to retention, the reduction in error was greater in MP (37 % or 6.7°) compared with AC (21 % or 4.2°). The control group had an error of 14.8° (±2.0°) at baseline and showed a borderline time effect (*p* = 0.056). Error decreased by 2.8° due to familiarization with the task and increased 0.1° 24 h later at retention.

There was a group × time interaction in the performance of the Purdue pegboard test [*F* (2, 40) = 8.3, *p* = 0.001]. Pegboard performance did not improve in MP (baseline, 13.3 ± 1.2 pins; after motor practice, 13.6 ± 1.4 pins; retention, 13.5 ± 1.4 pins). AC compared with MP placed 1.5 more pins on the board at the retention test (baseline, 13.6 ± 1.9 pins; after template viewing, 14.9 ± 1.8 pins; at retention, 15.0 ± 2.1 pins). Pegboard performance was stable in the control experiment (baseline, 13.0 ± 2.6; posttest, 13.4 ± 3.1; retention, 13.6 (±2.3) pins; *p* = 0.41).

### Peripheral nerve stimulation

Supramaximal stimulation of the radial nerve consistently evoked a Mmax with similar peak-to-peak amplitudes at baseline (MP, 2.4 ± 0.75 mV; AC, 2.1 ± 0.78 mV), after interventions (MP, 2.4 ± 0.89 mV; AC, 2.3 ± 0.74 mV), and at retention (MP, 2.4 ± 0.74 mV; AC, 2.3 ± 0.79 mV), resulting in no group × time interaction (*p* = 0.541) or a time main effect (*p* = 0.623). There was also no main effect of time in the control group (baseline, 2.7 ± 1.9; posttest, 2.7 ± 2.1; retention, 2.3 ± 1.3 mV; *p* = 0.465).

### Brain stimulation data

Table 2 shows the resting and active motor threshold and the corticospinal excitability data at rest and during the visuomotor task normalized and not normalized for Mmax and corticospinal excitability data during an isometric wrist extension at 20 % MVC normalized for MEP amplitudes in rest. The group × time interactions and the time main effects were not significant for RMT, AMT, and corticospinal excitability at rest and during the visuomotor task (all effects *p* > 0.05). However, there was a group × time interaction for contralateral facilitation measured as the facilitation of a standard motor evoked potential delivered at 120 % of RMT during a wrist extension at 20 % isometric MVC [*F* (2, 40) = 7.6, *p* = 0.002, see Table 2]. Facilitation was similar at baseline [MP, 340.7 % ± 148.7; AC, 386.3 % ± 159.9, *p* > 0.05). These data mean that the wrist extension at 20 % MVC facilitated the MEP measured at rest by 3.4- and 3.8-fold in MP and AC, respectively. Motor practice increased this facilitation to 400.2 % (±187.0), while the facilitation decreased to 329.2 (±109.5) in AC (both *p* < 0.05). At retention, the facilitation further increased in MP (627.0 ± 364.8) and further decreased in AC (292.2 % ± 106.6) (both *p* < 0.05). The difference in contralateral facilitation was 71 % after the intervention and 335 % at retention, with the facilitation being higher in MP vs. AC (*p* < 0.05). Thus, corticospinal excitability during a wrist extension at 20 % isometric MVC increased in MP but decreased in AC.

**Table 2:** Effects of motor practice and attentional control on corticospinal excitability (CSE)

	Baseline, mean (±SD)	After intervention, mean (±SD)	At retention, mean (±SD)
RMT (% SO)			
Motor practice	54.2 (10.9)	55.6 (12.5)	56.0 (14.2)
Attentional control	51.0 (10.3)	51.4 (11.4)	52.8 (11.7)
AMT (% SO)			
Motor practice	50.4 (12.2)	45.6 (12.9)	51.2 (20.6)
Attentional control	47.8 (6.8)	47.3 (6.8)	46.8 (3.5)
CSE (mV)			
Motor practice	0.35 (0.29)	0.39 (0.30)	0.26 (0.25)
Attentional control	0.30 (0.24)	0.27 (0.14)	0.26 (0.10)
CSE (% Mmax)			
Motor practice	15.5 (11.4)	16.7 (15.4)	11.6 (10.8)
Attentional control	14.6 (9.7)	12.3 (5.9)	13.3 (3.7)
CSE task (mV)			
Motor practice	1.01 (0.41)	1.01 (0.34)	0.77 (0.41)
Attentional control	1.05 (0.47)	0.96 (0.32)	0.92 (0.43)
CSE task (% Mmax)			
Motor practice	47.6 (30.2)	47.4 (26.4)	34.7 (19.6)
Attentional control	55.0 (24.7)	45.8 (26.0)	43.5 (22.5)
CSE during 20%MVC (% MEP rest)			
Motor practice	340.7 (148.7)	400.2 (187.0)^a,b^	627.0 (364.8)^a,b^
Attentional control	386.3 (159.9)	329.2 (109.5)^b^	292.2 (106.6)^b^

*RMT* resting motor threshold, *AMT* active motor threshold, *%SO* percent of stimulator output

^a^Group × time interaction (*F* (2, 40) = 7.6, *p* = 0.002)

^b^Facilitation increased in MP and decreased in AC relative to baseline with facilitation higher in MP than in AC after interventions and also at retention (all *p* < 0.05)

**Fig. 3:**
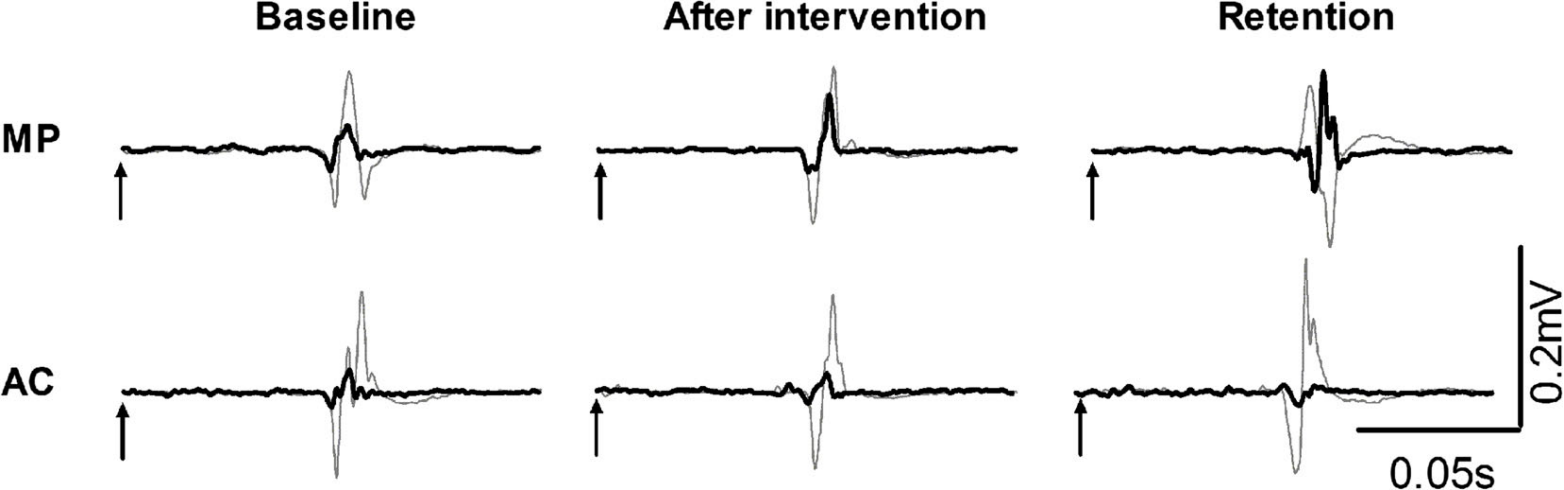
Representative responses to transcranial magnetic stimulation in the right extensor carpi radialis muscle for one 68-year-old female subject in the motor practice and in one 70-year-old female subject in the attentional control group. Recordings were made at rest at baseline, after intervention, and at retention. Waveforms represent average of five motor evoked potentials in response to single test pulses (*thin gray line*) and conditioned pulses (*thick black line*) at an interstimulus interval of 2 ms. *Arrows* indicate when the test pulse is given

**Fig. 4:**
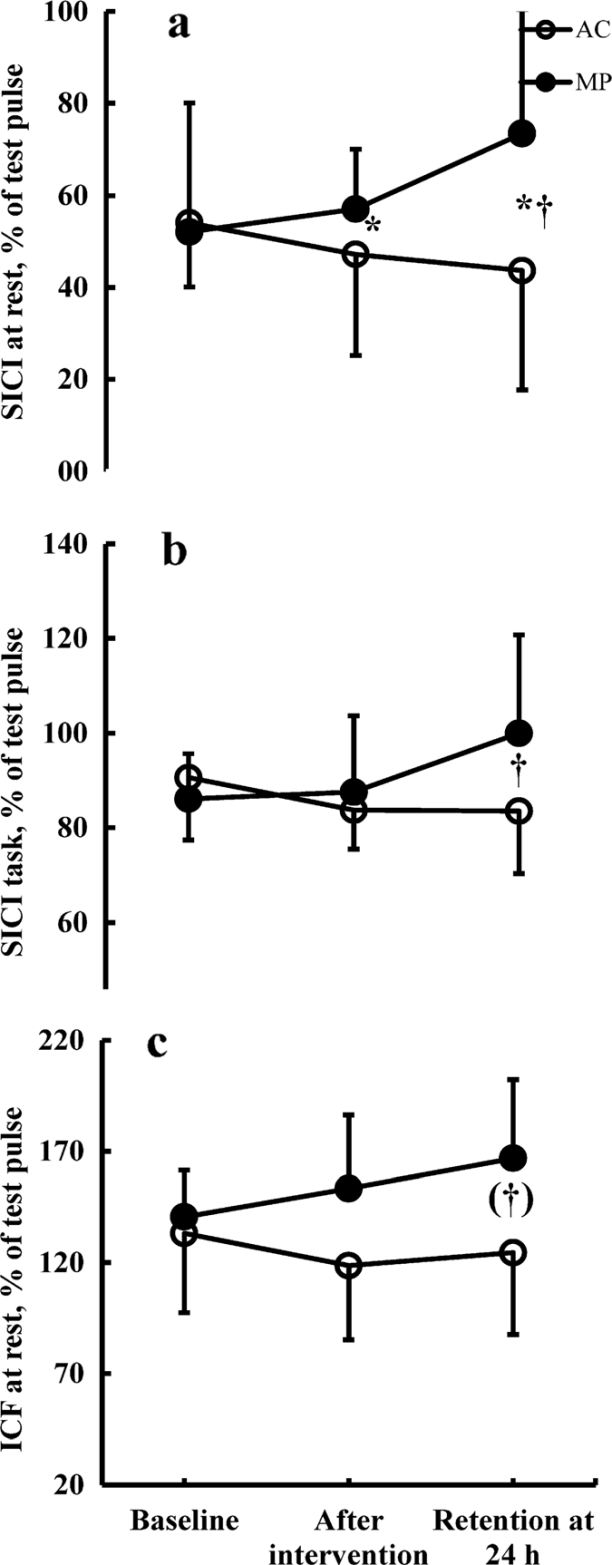
Effects of motor practice and attentional control on short-interval intracortical inhibition at rest (**a**), measured during the task (**b**), and intracortical facilitation measured at rest (**c**). **a** Group × time interaction [*F* (1.488, 28.272) = 4.6, *p* = 0.027]. *P < 0.05 between groups and ^†^ *p* < 0.05 relative to baseline. **b** Group × time interaction [*F* (2, 40) = 4.0, ^†^ *p* = 0.026]. **c** Borderline group × time interaction [*F* (2, 40) = 3.1, ^†^ *p* = 0.054]. SICI values <100 % indicate inhibition, and ICF values >100 % indicate facilitation. *Filled and open symbols* represent motor practice and attentional control, respectively. *Vertical bars* denote ±1 standard deviation

Figure 3 shows representative examples of SICI measured at rest in one subject in MP and AC subject, and Fig. 4 shows the group data of SICI and ICF. Figure 4a shows the group × time interaction for SICI recorded at rest [*F* (1.488, 28.272) = 4.6, *p* = 0.027]. The value of SICI was 52.1 % (±28.0) and 54.1 % (±14.0) in MP and AC, respectively, at baseline. After the interventions, the corresponding values in MP and AC were 57.1 % (±13.0) and 47.2 % (±22.0) (*p* < 0.05). After the interventions, nine of 11 subjects had less intracortical inhibition in MP, and nine of 11 subjects had more intracortical inhibition in AC. At retention, SICI was 73.5 % (±27.7) in MP and 43.7 % (±26.6) in AC (both between-group differences and relative to baseline *p* < 0.05). At retention, 10 of 11 subjects had less intracortical inhibition in MP, and 8 of 11 subjects had more intracortical inhibition in AC. Thus, intracortical inhibition decreased after MP, but it increased after AC.

Figure 4b shows the group × time interaction [*F* (2, 40) = 4.0, *p* = 0.026] for SICItask. As expected, the baseline values of SICItask were higher (88.4 % ± 11.4) than SICI (53.1 % ± 21.0), suggesting lower intracortical inhibition during contraction. The mean background EMG activity in the right ECR was 7.2 % (±3.2, MP) and 5.7 % [±2.7, AC, *t* test (20) = 0.83, *p* = 0.237] of the EMG activity measured in the ECR during a maximal effort isometric wrist extension. With similar SICItask values at baseline (MP, 86.1 ± 9.6; AC, 90.6 ± 13.2), the value of SICItask remained unchanged after MP (87.5 % ± 16.2) but decreased after AC (83.7 % ± 8.2). At the retention test, the value of SICItask increased in the MP group to 100.0 % (±20.8), while it remained the same in AC (83.5 % ± 13.3), resulting in a between-group difference of 16.5 % in the value of SICItask at retention (*p* < 0.05). Thus, intracortical inhibition decreased in MP and increased in AC both at rest and during the task, with the difference being especially prominent at retention.

We also measured the contralateral silent period during wrist extension at 20 % MVC. There was no group × time interaction [*F* (2, 40) = 1.7, *p* = 0.200] or a time main effect [*F* (2, 40) = 1.9, *p* = 0.163]. Pooled across the three time points, the average duration of the net silent period was 75.5 ms (±22.7) in MP and 71.0 ms (±16.5) in AC (*t* test: *p* = 0.368, data not shown).

Figure 4c shows the borderline group × time interaction for intracortical facilitation measured at rest [*F* (2, 40) = 3.1, *p* = 0.054]. The two groups were similar at baseline (MP, 140.6 % ± 20.9; AC, 133.2 ± 35.7), but ICF tended to increase in MP (153.3 % ± 33.0) and decrease in AC (118.6 % ± 33.4), a trend that continued at the retention test in MP but not in AC (MP, 166.9 % ± 35.4; AC, 124.5 % ± 36.9). ICFtask did not change (group × time interaction, *p* = 0.181, data not shown).

The control experiment revealed no time main effects for any of the TMS variables with the *p* values for the one-way repeated measures ANOVAs ranging from *p* = 0.143 to *p* = 0.874 (detailed data not shown).

### Correlation analyses

**Fig. 5:**
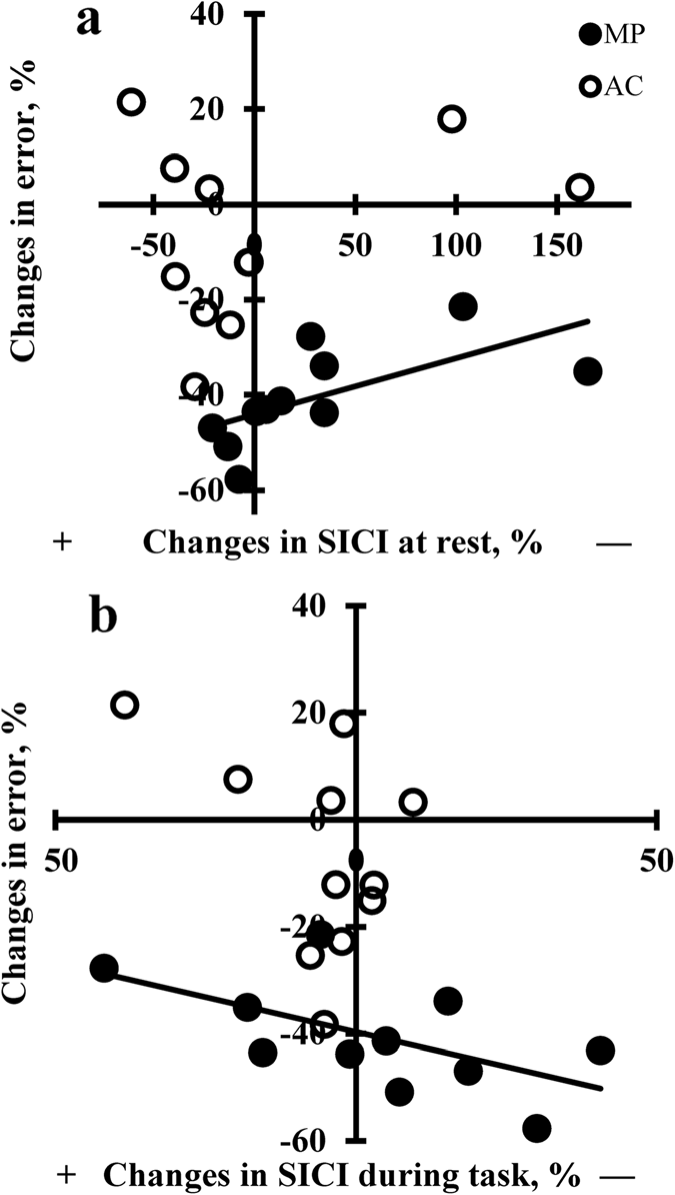
Correlation between percent changes in intracortical inhibition (*SICI*) and visuomotor skill in the motor practice group (*filled symbols*) and attentional control group (*open symbols*). Correlations are shown between **a** changes in SICI values at rest and changes in error (MP: *R* ^2^ = 0.41, *y* = 0.12*x* − 44.2; AC: *R* ^2^ = 0.08, *y* = 0.08*x* − 6.7), and **b** changes in SICI values during task and changes in error (MP: *R* ^2^ = 0.34, *y* = −0.26*x* − 39.7; AC: *R* ^2^ = 0.18, *y* = −0.61*x* − 10.3). The *positive* and *negative sign* denotes, respectively, more or less inhibition

Baseline levels and changes in visuomotor task and in the Purdue pegboard test did not correlate in MP, AC, and in the two groups combined (21 *r* values, *p* > 0.05). Changes in SICI measured at rest positively correlated with learning in MP (*r* = 0.64, *p* < 0.05) but not with the changes measured at retention (*p* > 0.05) (Fig. 5a). In contrast, changes in SICItask in MP negatively correlated with learning (*r* = −0.59, *p* < 0.05) but not with the changes measured at retention (Fig. 5b). These results indicate that an increased motor performance in MP is associated with more intracortical inhibition at rest and less intracortical inhibition during the task. None of these correlations were significant in AC.

## Discussion

We observed 40 % motor learning after only 20 min of practice of a visuomotor task, a skill that naive healthy old adults were able to consolidate into motor memory 24 h later. In contrast, watching the same templates without actual movements produced no learning (6 %, n.s). Corticospinal excitability at rest and during the visuomotor task remained unchanged in MP and AC but became strongly modified when measured during 20 % MVC. Intracortical inhibition at rest and during the task decreased, and facilitation at rest increased after MP. TMS metrics changed in the opposite direction in AC. Only in a few of these metrics did the changes correlate with changes in behavior. The findings partially support the global hypothesis that neuronal measurements in an active state vs. at rest are more selective and sensitive to motor learning and retention. We discuss the data in the context of how motor cortical disinhibition may play a key role in motor learning and motor skill consolidation in the healthily aging motor cortex.

### Skill acquisition

Old adults are normally able to learn a novel motor task. However, when compared with young adults, the results can be inconsistent as learning can be similar (Cirillo et al. 2011; Roig et al. 2014), become compromised (Coats et al. 2014; Rogasch et al. 2009; Swinnen 1998; Zimerman et al. 2013), or can even exceed young adults’ scores (Brown et al. 2009). Using models of error-based, reinforcement, and use-dependent learning (Wolpert et al. 2011), previous studies in healthy old adults reported 17–124 % learning (Cirillo et al. 2011; Coats et al. 2014; Rogasch et al. 2009; Roig et al. 2014; Seidler and Noll 2008; Seidler 2010), reflecting the fast phase of motor learning (Dayan and Cohen 2011; Luft and Buitrago 2005). The 40 % learning after just 20 min of motor practice in the present study is well beyond the 24 % reported in similar subjects, learning task, and exposure duration (18 min) but assessed in the index finger (Cirillo et al. 2011) (Fig. 2). Perhaps our task was more complex and represented a higher motor challenge compared with the finger (Cirillo et al. 2011) and therefore had more room for improvement. We note that, even though the 40 % learning exceeds learning rates reported in this study (Cirillo et al. 2011), it is possible that there was actually even greater learning in MP because 20 min of motor practice can cause a saturation effect and mask a portion of learning (Brawn et al. 2010; Rickard et al. 2008). Previous studies reported ∼24 % learning after ∼22 min of template tracking task in the finger (∼24 %, 18 min) (Cirillo et al. 2011), ankle (∼35 %, 32 min) (Perez et al. 2004), and elbow joint (∼12 %, 16 min) (Jensen et al. 2005) in young adults, suggesting that our old adults acquired the skill at the wrist as well if not more proficiently than young adults. This finding qualitatively agrees with previous studies (Brown et al. 2009; Cirillo et al. 2011) but warrants some caution because there is a growing concern that the young–old comparisons are misleading or even invalid when the baseline values are different in the two age groups, a factor that also guided our choice of experimental design (Vallence and Goldsworthy 2014). Another complicating factor that warrants caution is that the difficulty of the task templates in the current study differed from previous research. The large amount of learning did not transfer to a task variant because pegboard scores remained unchanged, and the changes in the learned and the transfer task did not correlate (*r* = 0.14, n.s.). We suspect that transfer did not occur because the learning exposure was too short and early learning processes, albeit engaged in transfer, act ineffectively over such a time scale (Seidler 2010) and because placing the pins requires movements around all three axes of the wrist joint and of the fingers while the learning task was confined to wrist movements in the transverse plane and excluded the fingers. Overall, our data provide evidence that healthy old adults retain the ability to acquire a novel visuomotor skill with high proficiency using wrist flexion–extension but with a low generalization to a task variant.

### Neuronal mechanisms of skill acquisition

Although we observed 40 % motor learning after motor practice and no learning as a result of visually following the same templates on the computer screen, a global measure of neuronal excitability, resting (53 % stimulator output) and active (49 % stimulator output) motor threshold, and a marker of use-dependent plasticity, i.e., MEP size at rest (0.33 mV) and during the execution of the task (1.03 mV), remained all unchanged (Table 2). Most often a lack of change or a reduction in MEP size after motor practice is interpreted as evidence for aberrations in long-term potentiation-like mechanisms involved in experimentally induced and use-dependent motor memory formation in aging humans (Fathi et al. 2010; Freitas et al. 2011; Muller-Dahlhaus et al. 2008; Sawaki et al. 2003; Todd et al. 2010). While age can certainly compromise M1’s ability to reorganize in response to motor practice (Boyd et al. 2008; Burke and Barnes 2006), we favor the interpretation of our MEP data to simply signify a dissociation between learning and one particular measure of plasticity. While short-term error-based visuomotor learning tends to increase MEP size in young adults (Coxon et al. 2014; Jensen et al. 2005; Perez et al. 2004), a dissociation was also reported in young subjects performing an interleaved form of motor practice (Coxon et al. 2014) and also in old adults who improved ballistic thumb abduction performance by 124 % but without changes in MEP size (Rogasch et al. 2009). As in the present study, learning outcomes after index finger practice also did not correlate with changes in MEP size in young and old adults (Cirillo et al. 2011). In young subjects, such associations were also not reported or found after one session of visuomotor practice in the ankle (Perez et al. 2004) and elbow joint (Jensen et al. 2005) and under certain conditions of serial reaction time task learning in the index finger (Tunovic et al. 2014). Even after 13 sessions of visuomotor elbow joint practice, associations were not higher than *R*^2^ = 0.236 (Jensen et al. 2005). It is possible that TMS accessed a different population of cells within the corticospinal path than the ones that were active during learning, an interpretation supported by animal data describing task-specific and selective activation of corticospinal neurons (Cheney and Fetz 1980; Muir and Lemon 1983). Compared with previous motor learning studies, we increased the specificity of the corticospinal measurements by assessing in old adults for the first time MEP size during the task itself but, as at rest, found no adaptations in this metric either, an observation that was not consistent with our hypothesis. However, when the contraction was stronger (20 % MVC) than during the task (5–7 % MVC), corticospinal excitability assessed by the contralateral facilitation test increased from 340 % (±148.7) to 400 % (±187.0) in MP and decreased in AC (*p* < 0.05, Table 2), data that are compatible with the hypothesis.

Because muscle contraction ≥20 % MVC compared with rest and weak contractions nonlinearly increase the magnitude and number of descending volleys during TMS, the contralateral facilitation data reflect how motor practice modified the contributions of the different early-phase I waves to the MEP (Di Lazzaro et al. 1998a). With contraction, adaptations most likely occurred through a summation of I1 and I2 waves. At rest and during weak contractions, a summation of I1–I4 wave is needed to produce MEPs (Di Lazzaro et al. 1998a; Smith et al. 2011). These data suggest that adaptation in specific portion of the corticospinal neurons occurred when corticospinal excitability is tested at 20 % MVC. The increased MEP at 20 % MVC in MP could also reflect a modulation of the input–output gain of individual motoneurons or at the level of the motoneurons pool (Kernell and Hultborn 1990). Collectively, the single-pulse TMS data suggest that, except for adaptations at stronger background contractions, indices of corticospinal excitability at rest and during the task were, in contrast with the hypothesis, under the present experimental conditions perhaps not sensitive, selective, or specific enough to detect changes normally used to index use-dependent plasticity after motor learning.

Intracortical inhibition at rest and during the task decreased and facilitation at rest increased after motor practice, but these outcomes changed in the opposite direction after the attentional control intervention (Fig. 4). SICI is a GABA-A-mediated inhibition that occurs in M1 circuits particularly affecting I3 waves (Di Lazzaro et al. 1998b; Kujirai et al. 1993), and, as demonstrated in slices prepared from the rodent primary motor cortex (Castro-Alamancos et al. 1995; Hess and Donoghue 1994), its reduction is associated with the induction of long-term potentiation, a process involved in motor learning (Floyer-Lea et al. 2006; Stagg et al. 2011).

In humans, intracortical inhibition indexed with SICI has, however, revealed somewhat inconsistent changes after motor practice: It decreased (Cirillo et al. 2011; Coxon et al. 2014; Gallasch et al. 2009; Garry et al. 2004; Hikosaka et al. 2002; Hinder et al. 2011; Liepert et al. 1998; Perez et al. 2004; Rosenkranz et al. 2007) or remained unchanged in young and old subjects (Cirillo et al. 2010; Rogasch et al. 2009). While corticospinal excitability data obtained through our single-pulse experiments increased only during 20 % MVC in MP (Table 2), our double-pulse SICI data at rest and during task agree with the trend for disinhibition acting as a mediating mechanism of improved performance after motor practice in old adults. The moderate negative association (*r* = −0.59, *p* = 0.043) between increase in motor performance and decrease in inhibition measured during the task assigns, as hypothesized, a functional role to disinhibition measured at least during the task (Fig. 5b). However, the direction of this association was positive at rest (*r* = 0.64, *p* < 0.034, Fig. 5a), suggesting a different role or involvement of these circuits at rest than during the task, a finding that future studies will have to confirm. Based on the current data, we are unable to disentangle whether the reduction in SICI measured during the task in MP is the result of a reduction in cortical GABAergic inhibition or a superimposition of a concurrent facilitation recruited during task contraction (Ortu et al. 2008). Because our recording conditions (5–7 % MVC during the task, 2-ms interstimulus interval, conditioning pulse of 70 % AMT) were similar under which previously “pure” SICI was identified, we favor the interpretation that a superimposition of short-interval intracortical facilitation on SICI played a small or no role in the SICI reductions in MP (Ortu et al. 2008) (Fig. 4a, b). We also note that neither intervention affected ICF during the task, and there was only a borderline group × time interaction at rest driven by the retention not the postintervention data (cf. Perez et al. 2004, Fig. 4c), suggesting a putative role for reduced GABA-A inhibition instead of facilitatory mechanisms mediating motor learning under these conditions. A lack of changes in contralateral silent period, a measure of GABA-B function (Chen 2004), further highlights the GABA-A system involvement.

### Skill retention

A few studies in old adults examined the retention of a learned skill 24 h after practice, using models of error-based, reinforcement, and use-dependent learning (Brown et al. 2009; Nemeth et al. 2010; Nemeth and Janacsek 2011; Roig et al. 2014; Swinnen 1998; Zimerman et al. 2013) but none with the template-matching error-based model. The pattern of no additional improvement but stabilization of the learned skill in the present study qualitatively agrees with the −10 to 10 % 24-h change reported in these studies (but see Coats et al. 2014). While motor skill acquisition occurs online, stabilization, and further improvements in the skill, and a reduction in the fragility of the motor memory traces are the results of offline processes (Dudai 2004; Fischer et al. 2005; Korman et al. 2003; Robertson 2009; Walker et al. 2003) that allow the consolidation of the skill into motor memory (Doyon and Benali 2005; Muellbacher et al. 2002; Robertson et al. 2004). Sleep can affect motor memory consolidation induced by error-based explicit motor learning under some (Walker et al. 2003) but not all conditions (Brawn et al. 2010). The quantity and quality of sleep was similar in MP and AC, making it unlikely that differences in these measures of sleep would have caused the observed differences in motor learning, retention, and neuronal excitability between the two groups.

Several of the TMS metrics revealed amplified changes at retention compared with the data after the interventions, recorded 24 h earlier. We are not aware any previous studies in healthy young or old adults reporting TMS data at 24 h after motor practice. During the offline period after the motor practice to retention, there was a continued reduction in SICI measured at rest and during the task and an increase ICF at rest (borderline) and strong additional increases in contralateral facilitation measured during 20 % MVC. The absence of correlations between the changes in these TMS metrics and learning outcome at retention suggest that memory trace stabilization was perhaps the result of neuronal processes other than the ones we measured, using the TMS metrics included in the study design (correlations not shown). This speculation is reinforced by the data seen in AC: There were significant improvements during offline period with a downward and opposite trend in the TMS metrics (Figs. 2 and 4). As in AC in the present study, finger-tapping practice in the sham control group in a previous tDCS study produced no learning, but performance increased at the 90-min retention test (Zimerman et al. 2013). However, the neuronal mechanisms that operate early after motor practice and mediate motor memory consolidation remain virtually unknown and require further studies (Dayan and Cohen 2011).

### Attentional control

The interaction in learning scores between MP and AC suggests that attention to visual elements and contextual cues of learning did not produce learning per se but affected learning outcomes at 24 h (16 % post-to-retention in AC, Fig. 2). Thus, the improvement in score at retention in AC must have occurred offline and was caused by a familiarization effect and/or cognitive processes. Because even after adjusting for learning due to familiarization with the motor task and repeated testing, there was still 1.5° less net error in AC compared with the control group, the possibility exists but requires further confirmation that the offline learning at retention in AC was related to procedural elements of the task. Processing of auditory, tactile, and visual information, as in the present study, can affect motor learning, as can cognitive processes such as attention to task details (Seidler 2010; Wolpert et al. 2011). Error-based learning engages the basal ganglia thalamocortical loops, medial cerebellum, the anterior cingulate cortex, the inferior frontal gyrus, and visual and parietal cortical, structures associated with cognitive aspects of the task, such as error detection and correction, working memory, and attention (Dayan and Cohen 2011; Hikosaka et al. 2002; Seidler and Noll 2008; Seidler 2010). More specifically, Thomson et al. (2008) reported that spatial attentional load but not variation in intensity of attention associated with dual tasking reduced SICI between successive responses of an index finger abduction task (Thomson et al. 2008). These results are in contrast to our data showing increase in SICI in AC (Fig. 4a, b). Thus, it remains unclear if recalling and anticipating the encoded visual cues associated with the motor task contributed to the improved performance at retention 24 h after the learning bout in AC.

It is possible that subjects in AC imagined themselves making the movement required for the visuomotor task, although we gave no such instructions. In this regard, our results are in agreement with the findings of a previous study (Debarnot et al. 2009), reporting motor performance gains in young individuals as a result of motor imagery after sleep. This interpretation is complicated by data suggesting that the age-related decline in motor imagery is more severe in complex motor tasks and tasks in laboratory settings compared with simple motor tasks and real-life settings (Kalicinski et al. 2015). Furthermore, studies have shown decreased inhibition after motor imagery, similar to executing real movements (Kumru et al. 2008; Liepert and Neveling 2009). In our study, the task was complex and motor cortical inhibition increased in AC. It is therefore unlikely that the AC group imagined making the movement required for the task.

### Limitations

Our design prevents us from drawing any inferences as to how motor performance, retention, and the neuronal mechanisms would compare with those in young adults. However, baseline differences between two age groups in motor performance complicate the interpretation of learning and retention data in numerous previous studies using the young–old comparison design (Vallence and Goldsworthy 2014). Although we measured corticospinal excitability at rest, during the task, and during 20 % contraction to assess adaptations in corticospinal excitability, taking one point on a nonlinear recruitment curve poses limits to our data and restricts the scope of interpretation. Furthermore, we only measured the Mmax at rest, which limits the interpretation of the corticospinal excitability data during the task. It is well established that fast motor learning involves not only M1, the only structure we probed, but also the networks that include the supplementary motor area, premotor cortices, and dorsolateral premotor cortex (Hikosaka et al. 2002; Petersen et al. 1998b; Sun et al. 2007). We did not quantify the effects of the two interventions on attention, but a previous motor learning study reported no effects on fatigue and attention (Zimerman et al. 2013). We did not examine any potential adaptations at the spinal level, but considering recent data from TMS-conditioned H-reflex paradigms, it is unlikely that H-reflex and F-wave measurements could have provided a definitive answer (Leukel et al. 2015; Taube et al. 2014). Finally, we acknowledge the limitation of performing a high number of comparisons, increasing the likelihood of type I error in some of our analyses.

### Conclusions

We observed 40 % motor learning after just 20 min of practice of a visuomotor task, a skill that naive healthy old adults were able to consolidate into motor memory 24 h later. The skill, however, did not transfer to a task variant. In contrast, watching the same templates without actual movements produced no learning. Corticospinal excitability at rest and during the task did not change but strongly increased during 20 % MVC in MP. Intracortical inhibition at rest and during the task decreased and facilitation at rest increased in MP. TMS metrics changed in the opposite direction in AC. The within-group changes and between-group differences were especially profound at retention administered 24 h after the two interventions. Motor cortical disinhibition as inferred from changes in SICI measured in the active muscle emerged as key mechanisms mediating learning and motor memory consolidation. The present results collectively suggest that the healthily aging motor brain can learn and retain a complex motor skill but may have some difficulty in transferring the acquired skill to a task variant. The results may also have relevance for the rehabilitation of old adults’ motor function compromised by neuronal injuries and disorders (e.g., stroke), requiring motor cortical reorganization through use-dependent plasticity.
